# Vinculin and filamin-C are two potential prognostic biomarkers and therapeutic targets for prostate cancer cell migration

**DOI:** 10.18632/oncotarget.19397

**Published:** 2017-07-19

**Authors:** Jianzhong Ai, Tao Jin, Lu Yang, Qiang Wei, Yang Yang, Hong Li, Ye Zhu

**Affiliations:** ^1^ Institute of Urology, West China Hospital, Sichuan University, Chengdu, Sichuan, P.R. China; ^2^ Department of Urology, West China Hospital, Sichuan University, Chengdu, Sichuan, P.R. China; ^3^ Department of Cardiology, West China Hospital, Sichuan University, Chengdu, Sichuan, P.R. China; ^4^ Animal Experimental Center, West China Hospital, Sichuan University, Chengdu, Sichuan, P.R. China

**Keywords:** VCL, FLNC, prostate cancer, migration, quantitative proteomics

## Abstract

Prostate cancer (PCa) is one of the most common diseases for male population, and the effective treatment for metastatic castration-resistant PCa is still lacking. To unravel the underlying mechanism of PCa cell migration, we plan to analyze the related crucial proteins and their roles. In our study, we firstly identify the differentially expressed proteins using quantitative proteomics, and confirm their mRNA expression using quantitative polymerase chain reaction (qPCR). The alterations of these proteins at DNA and mRNA levels are obtained from cBioPortal database. Furthermore, the functions of these proteins are evaluated using wound-healing assay. The quantitative proteomics identified vinculin (VCL) and filamin-C (FLNC) as two highly expressed proteins in PC3 cells, and the DNA and mRNA of these two proteins were amplified and upregulated in a part of PCa patients. Knockdown of VCL and FLNC gene expression significantly inhibit PCa cell migration. These findings suggest that VCL and FLNC identified by quantitative proteomics are highly expressed in PCa cells with high migration potential, and they could be effective targets for repressing PCa cell migration, paving a new avenue for the prognosis and treatment of advanced PCa.

## INTRODUCTION

As we know, prostate cancer (PCa) is now one of the most common malignancies among male population, it is also the second most diagnosed malignancy and fifth leading cause of cancer-related death worldwide [1]. For past decades, several critical drugs were developed for the treatment of PCa, particularly for advanced PCa, such as enzalutamide and abiraterone [2, 3]. However, the efficacy of these drugs were markedly limited by the relapse of PCa. Currently, the underlying mechanism for the relapse is still largely unknown. It is an effective way to inhibit the PCa progression by repressing its migration and metastasis. Hence, this study utilized the quantitative proteomics to unravel the crucial proteins tightly associated with the cancer cell migration.

Quantitative proteomics is an excellent technique for analyzing the differential expressed proteins among different cell lines and/or tissues [4, 5]. It is characterized by high throughput and accuracy. This technique plays very important roles in discovering potential drug targets, biomarkers and critical signaling pathway of diseases. For those above advantages, we employed quantitative proteomics to identify the important proteins that are responsible for PCa progression.

LNCaP and PC3 cells represent excellent cell models for PCa progression, they were derived from different sites of human patients with low and high migration potentials, respectively [6]. Our previous study demonstrated their differential capabilities, and identified the differential proteins, including VCL and FLNC [7].

Vinculin (VCL) is known as an actin filament-binding protein that was involved in cell-matrix adhesion and cell-cell adhesion, and it regulates the E-cadherin expression on cell-surface and potentiates mechanosensing by the E-cadherin complex [8]. Also VCL may play important roles in cell morphology and locomotion [8]. The complex that was composed of VCL can serve to anchor actin filaments to the membrane [9].

Filamin C (FLNC) has an N-terminal filamentous actin-binding domain, followed by a lengthy C-terminal self-association domain containing a series of immunoglobulin-like domains, and a membrane glycoprotein-binding domain [10]. Also it interacts with γ-sarcoglycan and δ-sarcoglycan at the sarcolemma [11]. FLNC is an important component of cytoskeleton, and it plays a role in cell migration and signal transduction [12].

In this study, we propose to analyze the differential proteins for PCa cell migration using quantitative proteomics, and further investigate the clinical significance of identified proteins using cBioPortal database. Finally, the roles of genes of interest in PCa progression were evaluated using functional assays. These findings will pave a way for developing novel prognostic biomarkers and therapeutic targets.

## RESULTS

### VCL and FLNC are highly expressed in PC3 cells

**Figure 1:**
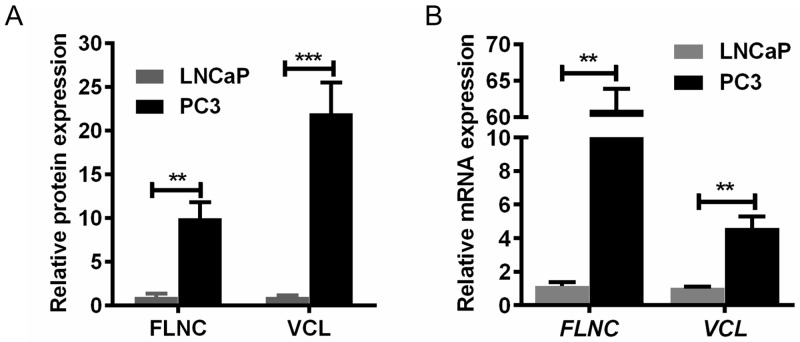
VCL and FLNC mRNA and protein expression in PC3 cells is higher than that in LNCaP cells **(A)** The protein levels of FLNC and VCL were approximately 10-fold and 20-fold overexpressed in PC3 cells. **(B)** The trend of FLNC and VCL mRNA expression was consistent with their protein overexpression. **, p<0.01; ***, p<0.001.

To analyze the underlying mechanism for distinguishable migration capabilities between LNCaP and PC3 cells, our previous study have employed quantitative proteomics to identify differentially expressed proteins [7]. As per the bioinformatics analysis, two highly expressed proteins in PC3 cells, VCL and FLNC, were implied to play important roles in PCa cell migration (Figure 1A), and the mRNA expression of these two proteins was further confirmed by qPCR (Figure 1B). These data suggested that VCL and FLNC presented significantly higher expression at mRNA and protein levels in PC3 cells than that in LNCaP cells, indicating its potential roles in PCa cell migration.

### VCL and FLNC present DNA amplification and mRNA upregulation in PCa patients

**Figure 2:**
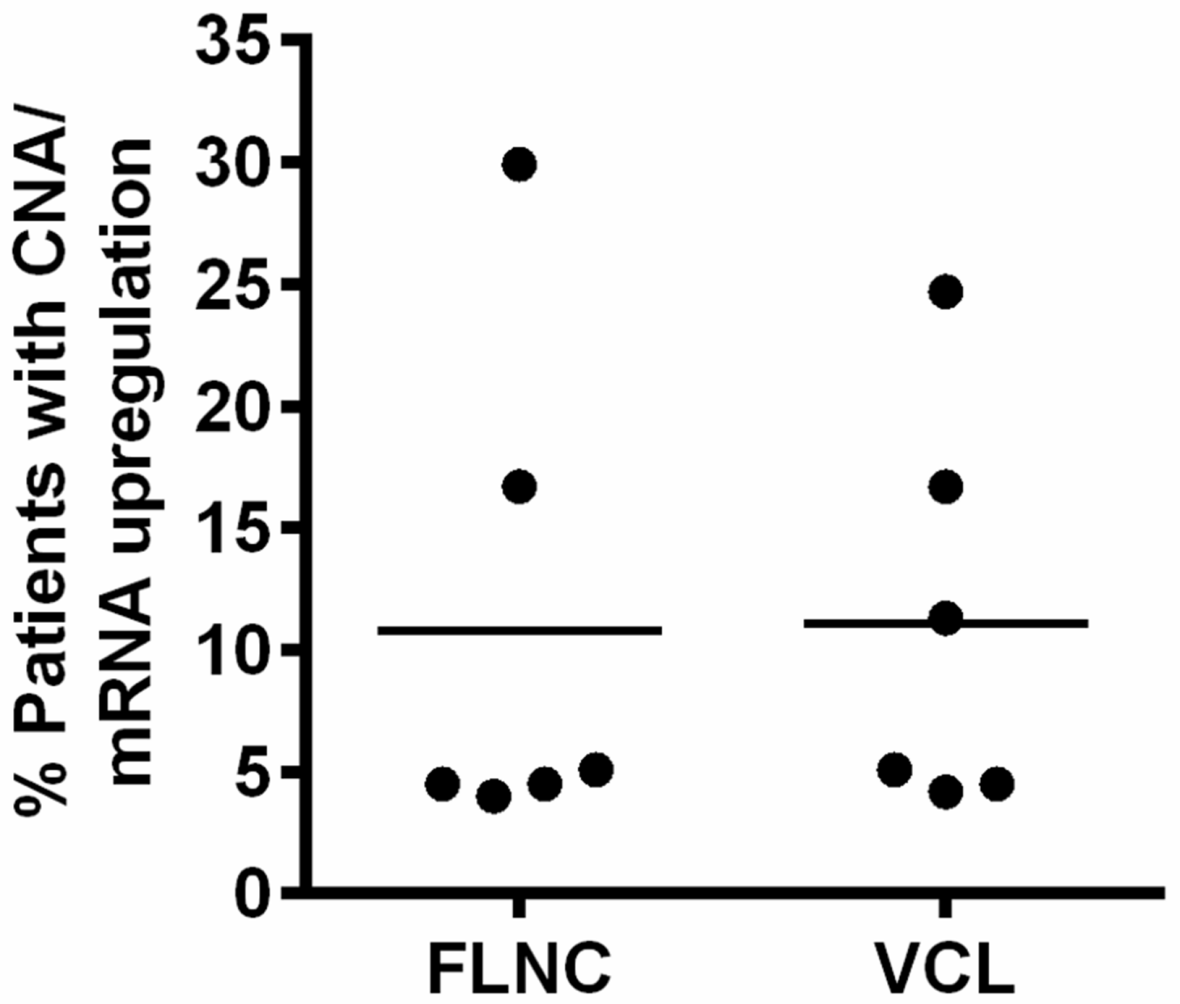
Alterations of FLNC and VCL CNA and mRNA in PCa patients The data were collected from independent cohort studies, the ratio was calculated using the following formula: %=Number of patients with CNA and/or mRNA upregulation/total patient number of a study. CNA, copy number amplification.

To further investigate the potential role or function of VCL and FLNC, we analyzed the conditions of DNA amplification and mRNA upregulation in PCa patients using cBioPortal database. As shown in Figure 2, the ratio of patients with DNA amplification and mRNA upregulation of VCL and FLNC ranged from 5% to 30% in different cohort studies (6 studies for VCL and 6 studies for FLNC). This finding indicated that VCL and FLNC indeed overexpressed in a part of PCa patients, and showed the clinical significance for studying the underlying mechanism of VCL and FLNC in PCa progression.

### VCL and FLNC expression is effectively downregulated by short-hairpin RNA

**Figure 3:**
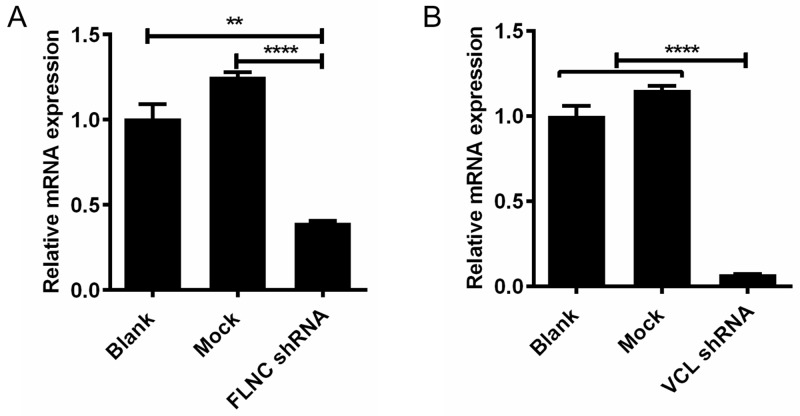
Knock-down of FLNC and VCL using shRNA **(A)** Significant downregulation of FLNC at mRNA level using its shRNA. **(B)** Significant downregulation of VCL at mRNA level using its shRNA. **, p<0.01; ****, p<0.0001.

To study the roles of VCL and FLNC in cell migration, we have constructed the shRNA plasmids and performed cell transfection. After 48 h of transfection, the mRNA expression of VCL and FLNC was assessed by qPCR. As illustrated in Figure 3A and 3B, the mRNA expression of FLNC and VCL was significantly decreased when compared with blank and mock control groups. Our data suggested that FLNC and VCL expression could be effectively repressed by the specific shRNAs.

### Downregulation of VCL and FLNC significantly inhibits PCa cell migration

**Figure 4:**
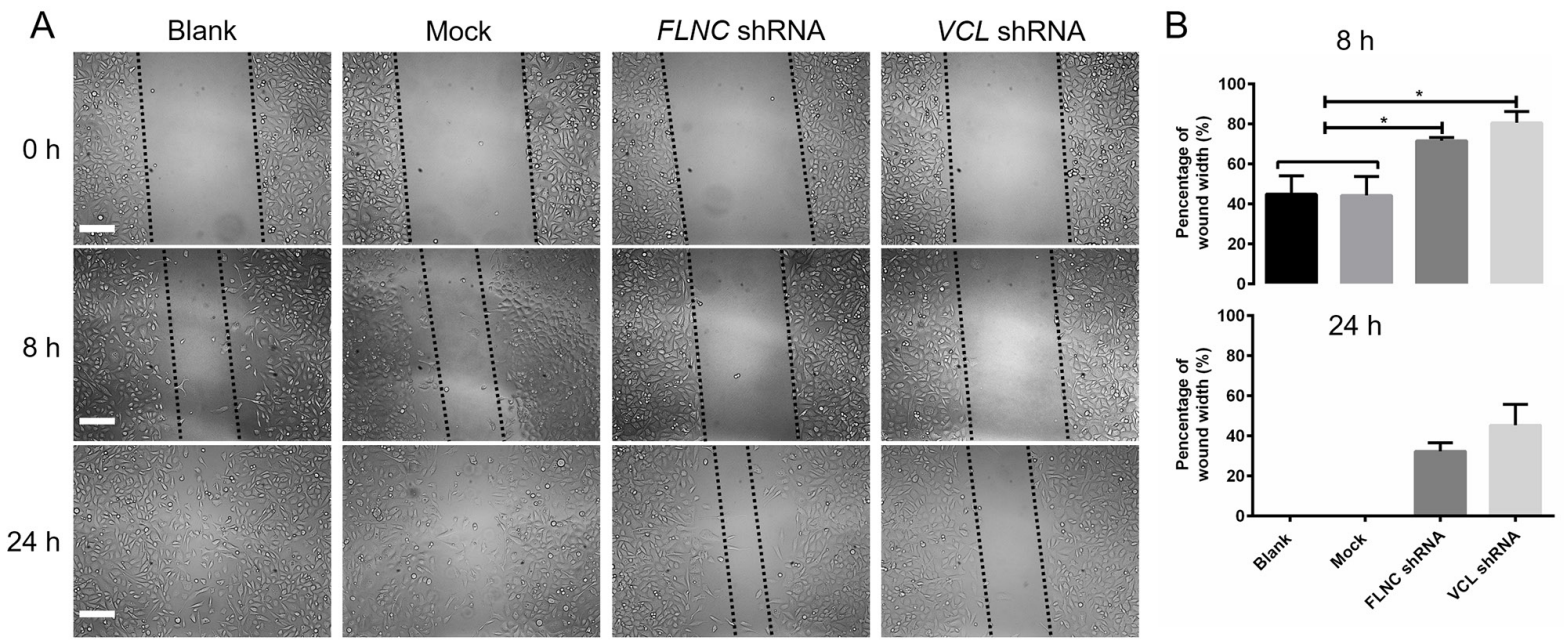
Detection of cell migration capabilities using a wound-healing assay **(A)** Cells were scratched using a 10-μL tip, and the wound widths were recorded at 0, 8 and 24 h post-scratch. **(B)** The widths were measured using image J software, and the data were analyzed using Prism 6.0. *, p<0.05.

To evaluate the potential role of VCL and FLNC in PCa cell migration, we have screened the cell line stably expressed VCL and FLNC, and the wound-healing assay was used to detect the migration capabilities. After 8 and 24 hours of scratch, the wound widths in the blank and mock control groups were significantly decreased when compared with FLNC shRNA and VCL shRNA groups (Figure 4A and 4B). These data showed that downregulation of VCL and FLNC markedly inhibited PCa cell migration, indicating the crucial role of these two proteins in PCa progression.

## DISCUSSION

PCa is one of the top malignancies for male population worldwide [1]. At present, PCa is the most common diagnosed malignancy, and the third cause of cancer-related mortality in USA [13]. Recently, the incidence of PCa is significantly increasing in Asian countries, including China and Japan [14]. Progression of PCa dramatically influenced male urinary and sexual functions, and decreased the quality of life [15]. Particularly, advanced metastatic PCa is accompanied with metastasizes to bone, lung and lymph node [16-18]. Currently, many methods were used to treat PCa, including surgery, local cryotherapy, castration, and chemotherapy [19, 20]. During the last decade, several new drugs have been developed, such as sipuleucel T, abiraterone and enzalutamide [2, 3, 21]. Those progresses had lighted the prospects of PCa treatment, however, PCa would relapse after a certain time of treatment using above drugs [22]. Hence, it is very critical for digging the underlying mechanism of PCa and developing more effective treatments. This will greatly be benefit to PCa patients by extending life span, relieving the pain and improving the quality of life.

Comparative proteomics have been developed for decades, and it performed very well in finding diagnostic biomarkers and drug targets [23, 24]. Also this technique is excellently useful for analyzing the underlying mechanisms of diseases. Our study utilized Q Exactive mass spectrometer to acquire high throughput and accuracy data, and these data were processed by proteome Discoverer and Mascot Server [7]. Finally, we successfully identified the differential expressed proteins, and these proteins could be the potential biomarkers for diagnosis or potential targets for drug developing. Furthermore, we have confirmed the differential expression of the interesting proteins at mRNA level. Our data showed that the mRNA levels of FLNC and VCL were not consistent with their protein levels, and this was probably caused by two reasons: 1. The differential proteins were identified by high throughput mass spectra analysis, hence, some deviations would be presented in the protein quantification; 2. Even the trend would be the same, however, the fold change of protein expression of a certain gene is not always consistent with fold change of mRNA expression. As described previously, those differential proteins were clustered as per their different biological functions [7]. This study focus on a cluster of proteins function as mediator of cell-cell or cell-matrix interactions or components of cytoskeleton. VCL and FLNC were two important proteins for cell migration, and they were indeed highly expressed in PC3 cells (Figure 1), a PCa cell line with high migration potential, as well as in PCa patients (Figure 2).

The patient data indicated the critical clinical significance of FLNC and VCL in the progression of PCa, suggesting that a part of patients would be benefit from the knockdown of these two genes. Based on these patient data, the *in vitro* experiments were designed to verify the roles of these two differentially expressed proteins in PCa progression. Accordingly, this study proposed to downregulate VCL and FLNC expression using corresponding shRNAs, the qPCR data showed the significant downregulation of VCL and FLNC. After that, wound-healing assay was used to evaluate the role of VCL and FLNC in PCa cell migration, and the findings obviously showed the inhibitory effect of VCL and FLNC shRNAs on cell migration.

Although our current data strongly suggest that these two proteins have a high potential to become prognostic biomarkers and therapeutic targets for PCa, further studies in *in vivo* models and PCa patients are critically needed before they can be used clinically. Our future studies will focus on detecting the expression of FLNC and VCL in patients with different stages of PCa. In addition, we will further investigate the role of FLNC and VCL in promoting PCa cell migration using mouse models.

In sum, our study performed a standard comparative proteomics study, we have accurately identified two important differential proteins by mass spectra, and investigated the clinical significance of these two proteins using a popular large-scale cancer genomics data sets. These data indicated the necessity for further functional study. Finally, our findings suggested that VCL and FLNC were highly expressed in PCa cells with higher migration capability and PCa patients, and they played key roles in PCa cell migration.

## MATERIALS AND METHODS

### Cell culture

293T cells (CRL-3216) and human PCa cell lines, PC3 (CRL-1435) and LNCaP (CRL-1740), were obtained from American Type Culture Collection (ATCC, VA, USA). PC3 and LNCaP cells were cultured in Dulbecco’s Modified Eagle’s Medium (DMEM, ATCC, VA, USA) and RMPI-1640 (ATCC, VA, USA) supplemented with 10% of fetal bovine serum (FBS, Life Technologies, NY, USA) in a humidified atmosphere of 5% CO_2_. In addition, 293T cells were cultured in DMEM medium supplemented with 10% FBS, 2mM L-glutamine (ATCC 30-2214), and 1% Penicillin/Streptomycin in a humidified atmosphere of 5% CO_2_.

### Quantitative proteomics analysis

The procedures of SDS-PAGE analysis, in-gel digestion and mass spectra analysis were performed as described previously [7].

### cBioPortal analysis

The alterations of DNA and mRNA levels of a certain gene can be obtained by searching cBioPortal database using the website of http://www.cbioportal.org/. The parameters of the database should be defined as follows: cancer study, genomic profiles, patient/ case sets and genes of interest. Then the data could be obtained one by one [25, 26].

### shRNA constructs and lentivirus packing

The shRNA sequences were inserted into pLKO.1 vector using NdeI and EcoRI restriction sites. The mature antisense sequences for VCL and FLNC are as follows: ATTTATTAGCAGTACCAACCG (VCL), and ATTGTTGGGAACCACCTTAGC (FLNC). For lentivirus packaging, the cell culture medium was replaced with 20 mL of fresh complete growth medium one hour prior to transfection. Next, the transfection mixture containing 2× HBS, Hepes, CaCl_2_, cis vector DNA and helper plasmids was prepared and incubated at room temperature for 20 min. Twenty-four hours later, the culture medium was replaced with OptiMEM medium supplemented with 1% Pen/Strep. The culture medium was harvested on day 3 and 4 post-transfection, and finally the culture medium containing lentiviruses was filtered using 0.45 micron syringe filter.

### Stable cell line screening

Firstly, 1×10^5^ cells were seeded into a 6-well plate, and the cells were cultured with 500 μL of viral supernatant, 9.5 mL of fresh medium and 10 μg of polynrene. The cell culture medium was replaced after 24 hours of infection. After 48 hours of infection, the cells were cultured with medium with puromycin and passaged continuously for 7-10 days.

### Real-time qPCR

Total RNA was extracted using Trizol reagent from ThermoFisher Scientific (MA, USA). The first strand cDNA was obtained from 1 μg of total RNA by reverse transcription using a high-capacity cDNA reverse transcription Kit (MA, USA). Next, the quantitative PCR (qPCR) was performed using the primers as follows: *VCL*, CTCGTCCGGGTTGG AAAAGAG (sense), AGTAAGGGTCTGACTGAAGCAT (antisense); *FLNC*, CAACGTG GATGAGCATTCTGT (sense), GTCCTCGATGTAGACCAGCAC (antisense); *ACTB*, CATGTACGTTGCTATCCAGGC (sense), CTCCTTAATGTCACGCACGAT (antisense). The PCR amplification profile was as follows: one cycle of 50°C for 2 min and 95°C for 10 min; 40 cycles of 95°C for 15 s and 60°C for 1 min. *ACTB* was used as an internal control.

### Wound-healing assay

This assay was performed as described previously [8]. Briefly, 3 × 10^5^ cells were seeded into a 6-well plate, and the complete culture medium was replaced with serum-free culture medium 6 h before the wound scratch. The mono layer cells were scratched using a 10-μL tip, and the cells were cultured with serum-free medium continuously. The images of healed wound at 0 h and 8 h were recorded using a common microscope, and the width of the wound was obtained using Image J software.

### Statistical analysis

All data were represented as means ± the standard deviation (SD). Statistical significances for comparisons between groups and among multiple groups (> 3) were determined using a Student’s paired t-test and analysis of variance (ANOVA), respectively, in Prism 6.0, and p < 0.05 was the level of significance.

## Abbreviations

VCL: vinculin
FLNC: filamin-C
PCa: prostate cancer
qPCR: quantitative polymerase chain reaction
